# Computational fluid dynamic characterization of carotid bifurcation stenosis in patient-based geometries

**DOI:** 10.1002/brb3.25

**Published:** 2012-01

**Authors:** Clemens M Schirmer, Adel M Malek

**Affiliations:** Cerebrovascular and Endovascular Division, Department of Neurosurgery, Tufts Medical Center and Tufts University School of MedicineBoston, MA

**Keywords:** Atherosclerosis, Carotid artery, Endothelium, Fluid mechanics, Stenosis

## Abstract

Hemodynamic forces play a role in determining endothelial cell (EC) phenotype and influence vascular remodeling. We present a lesion-based computational fluid dynamic (CFD) pilot analysis to understand the complex spatial and temporal hemodynamic changes that prevail in patients with high-grade carotid artery stenosis (CS). High-resolution three-dimensional (3D) rotational angiography datasets were acquired in eight patients, and used to generate computational meshes. CFD analysis was carried out implementing realistic shear-dependent viscosity for blood. The mean wall shear stress (WSS) within the stenosis region was 107 ± 73 dyn/cm^2^ rapidly followed by direction reversal and lower oscillating values in the recirculation zone at a mean of 19 ± 14 dyn/cm^2^. WSS vectors exhibited complex dynamic directional and amplitude oscillations not seen in healthy segments, along with time-dependent convergence and divergence strips during the cardiac cycle. The spatial gradient of WSS revealed an elevated average magnitude at the throat of the stenosis of 1425 ± 1012 dyn/cm^3^. In conclusion, patient-based CFD analysis of CS predicts a complex hemodynamic environment with large spatial WSS variations that occur very rapidly over short distances. Our results improve estimates of the flow changes and forces at the vessel wall in CS and the link between hemodynamic changes and stenosis pathophysiology.

## Introduction

Cervical carotid artery stenosis (CS) is diagnosed using a combination of history, clinical examination, and imaging. Rapid advancement of noninvasive imaging modalities notwithstanding, biplane and rotational digital subtraction angiography still provide unsurpassed anatomic resolution of the endoluminal aspect of CS. As the evaluation of angiographic images remains limited to the measurement of the geometric degree of stenosis, the ultimate evaluation of a stenosis relies on the experience of the treating physician. Computational fluid dynamic (CFD) methods can offer an additional important layer of functional information to enrich and complement the anatomical information.

A link between atherogenesis and wall shear stress (WSS) forces, defined as the internal friction forces between the flowing blood and the vessel wall, has been proposed, suggesting that high shear stress could lead to both mechanical damage to the endothelial cells (ECs) and potential denudation (Fry 1968, 1969). Low and oscillatory shear stress promote monocyte adhesion to the EC through the increased expression of vascular adhesion molecule-1 (VCAM-1), which bind integrins expressed on leukocytes and direct their firm adhesion to and entry into EC (Caro et al. 1971; Berger and Jou 2000). In contrast, atheroprotective flow activated Nrf2 and protected EC against oxidative stress injury (Dai et al. 2007).

Vessel geometry and hemodynamic forces are major regulatory factors of normal and pathologic vessel wall function in arteries (Berger and Jou 2000). Most studies in this field have been carried out in carefully controlled in vitro experimental setups, which do not reproduce the in vivo characteristics of blood flow through stenosed vessels.

Although CFD analysis has been applied to theoretical stenoses, detailed simulations that predict the spatial and temporal pattern of WSS within actual patient-based stenotic lesions are scarce because of computational complexity (Thomas et al. 2003) and a lack of high-resolution spatial data describing the three-dimensional (3D) geometry of atherosclerotic vessels.

## Patients and Methods

Eight patients (two female, median age 76 years) with symptomatic CS of the cervical carotid bifurcation recalcitrant to medical therapy were evaluated. Five lesions were located on the left side (63%). Three patients had ischemic symptoms at the time of evaluation. The patients underwent catheter-based digital-subtraction cerebral angiography in biplane and 3D rotational modes. Median degree of stenosis by ultrasonographic Doppler examinations performed in seven of eight patients was 95% and median angiographic stenosis was 88% by NASCET criteria (Nagel et al. 1991).

A detailed description of the CFD methods has been previously described (Schirmer and Malek 2007a, b). Briefly the 3D volumetric datasets, reconstructed from rotational angiograms, were segmented and used to generate hybrid, predominantly hexahedral, meshes with refinement zones over the area of the carotid bifurcation and the internal carotid (ICA). Computations were carried out using Fluent (Ver. 6.2.16, Fluent Inc, Lebanon, NH) on a cluster of parallel computers. A transient laminar flow model using a Carreau non-Newtonian formulation of the viscous properties of blood (Schirmer and Malek 2007a, b), nonslip and nonpenetration constraints at the wall were assumed for the simulations. Pulsatile CFD was performed for three cardiac cycles (Schirmer and Malek 2007a) with a 500 timestep pulsatile velocity waveform that was derived from waveforms described in healthy human subjects by Ford et al. (Holdsworth et al. 1999). Validation of the computational approach used in this study has been previously reported (Schirmer and Malek 2007a, b, 2008). Postprocessing was performed using Ensight software (Ver. 8, CEI, Apex, NC). Statistical analysis of mean values was performed using Student's *t*-tests and statistical significance was assumed for *P* < 0.05 (SAS, Cary, NC).

## Results

### Changes of the flow pattern in CS

**Figure 1:**
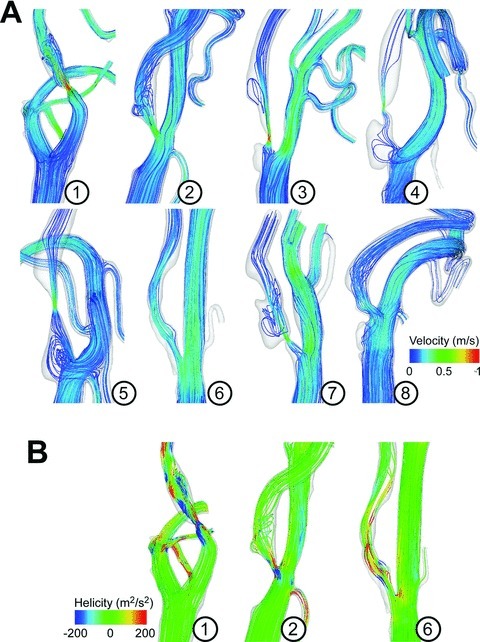

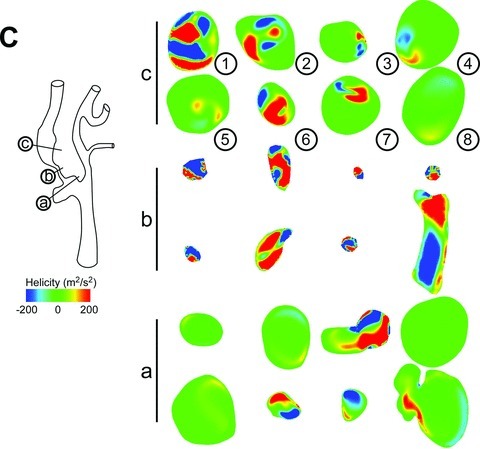
**(A)** Pathlines color coded for the time-averaged velocity magnitude. **(B)** Examples of pathlines, color coded for the helicity density. **(C)** Helicity density on orthogonal cutplanes that correspond to a prestenotic cutplane *a* (see schematic), a cutplane at the throat of the stenosis *b*, and a cutplane in the poststenotic region (PSR) *c*.

**Figure 2:**
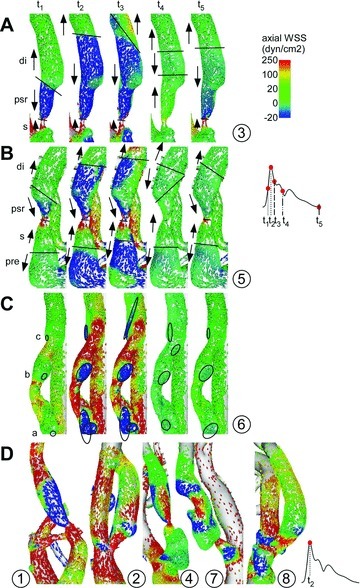
**(A–C)** Three example (case 3, 5, and 6) detailing the temporal evolution of the instantaneous wall shear stress (WSS) vectors at the stenosis and poststenotic region (PSR) during the cardiac cycle (reds points on pulse wave). Regions of antegrade or retrograde axial WSS direction (*pre*, *s*, *di*, *psr*, *di*) are demarcated by lines that demonstrate the stepwise changes of the location of the zone of shearreversal between these regions. WSS vortices are outlined (circles *a*–*c*) at the wall as it migrates in a cyclic fashion during the cardiac cycle. **(D)** Instantaneous WSS vectors at peak-systole in cases 1, 2, 4, 7, and 8.

Starting with laminar flow in the common carotid (CCA), a considerable distortion of the flow pattern was seen in all eight cases (Fig. 2A). The average Reynolds number in the stenosis was 114 ± 30, the maximum 162. Three modes of flow alteration were discerned as a function of the geometry of the stenosis: in axisymmetric stenosis of the ICA (cases 1 and 7) recirculation and secondary flow patterns was seen downstream from the stenosis in the poststenotic dilatation of the vessel. The jet of accelerated blood in the center of the vessel downstream of the stenosis evolves into increased twisting and curling of the flow, characterized by the pseudoscalar quantity helicity (Fig. 1B, cases 1 and 7). In cases where the stenosis was close to the carotid bulb or just downstream to the bifurcation (cases 3–5 and 8), recirculation developed both upstream and downstream to the stenosis. Significant twisting of the flow with increased helicity, however, could only be demonstrated on orthogonal cutplanes through the stenosis itself (panel b) and in the poststenotic segment of the flow (see insert panel a in Fig. 1C, cases 1–2 and 6–7). The increase in helicity is characterized by the development of a division of the pathlines into a right-handed and left-handed twisting component of the flow. One case with an elongated and flattened stenosis twisted around itself (case 6) had only small areas of recirculation, but increased helicity along the length of the stenotic vessel segment (Fig. 1C, case 6). The two corkscrew components of the flow merge further downstream upon restoration of a laminar flow condition (Fig. 1B, case 2, distal ICA).

### WSS in carotid stenosis

**Figure 3:**
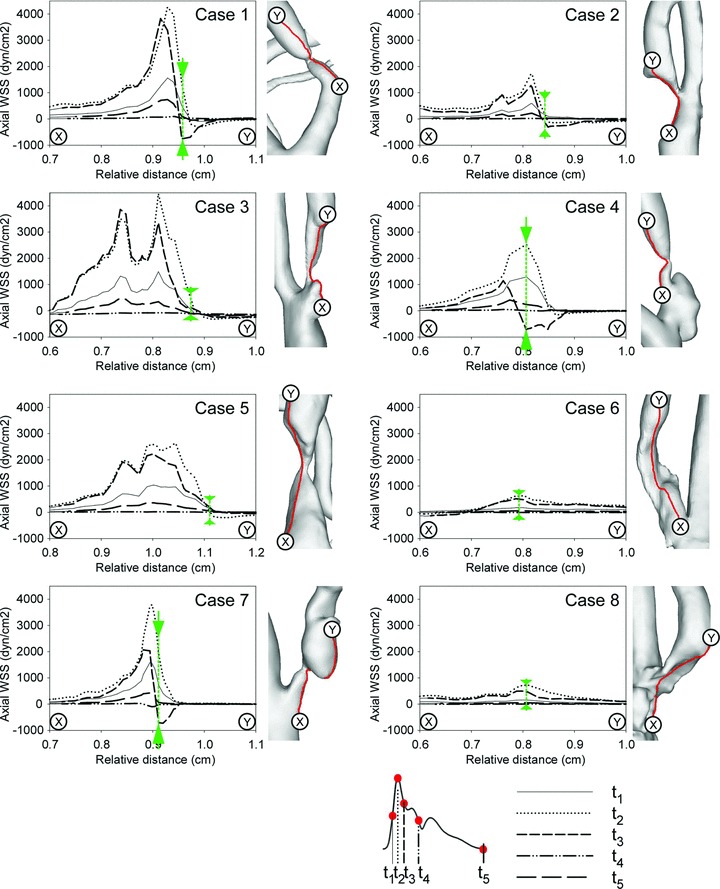
Axial wall shear stress (WSS) magnitude along the line between points X and Y (see schematic on right of each panel) during five points of the cardiac cycle. Green arrows demarcate the point of maximal shear reversal during the cardiac cycle.

**Figure 4:**
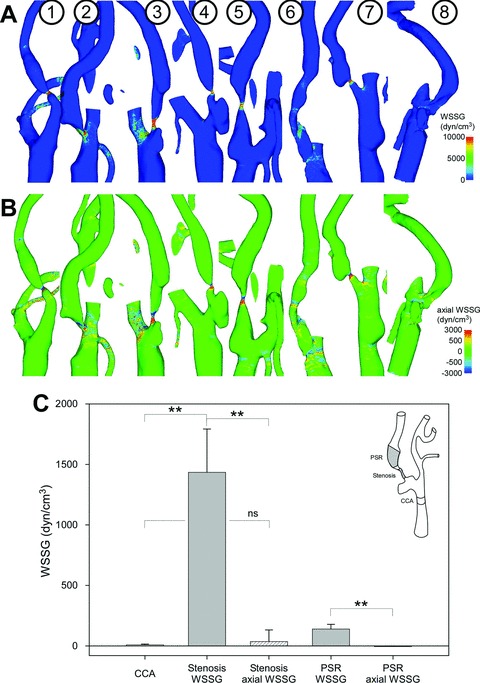

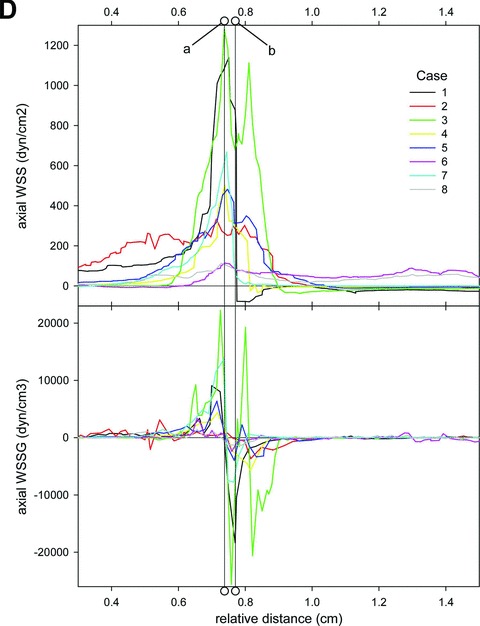
Contour plots of the wall shear stress gradient (WSSG) magnitude **(A)** and the axial WSSG component **(B)** averaged over the cardiac cycle. **(C)** Bargraph showing time-averaged and spatially averaged mean magnitudes of the WSSG and directional magnitudes of the antegrade WSSG in the distal CCA, at the stenosis and at the expansion of the poststenotic region (PSR). Values are mean ± SD, the insert shows the schematic position of the regions of interest. **(D)** Axial wall shear stress (WSS) magnitude (top graph) and axial WSSG magnitude along the line between points X upstream to the stenosis and point Y downstream of the stenosis (see Fig. 5), averaged over the course of the pulse cycle. Line *a* indicated the peak WSS and corresponding reversal of the axial WSSG direction. Line *b* indicates the secondary reversal band.

The WSS magnitude, averaged over the course of the cardiac cycle, was computed and exhibited a visible increase in the area of the stenosis (Fig. 2); in cases with concentric stenosis an increase of the axial component of the WSS was found (Figs. 3 and 4D). Three regions of interest were defined: the distal CCA, the stenosis proper, and the poststenotic region (PSR), where values were averaged spatially and temporally and over all cases in this study. The average WSS magnitude at the throat of the stenosis was 107 ± 73 dyn/cm^2^, significantly higher than 23 ± 11 dyn/cm^2^ in the healthy CCA segment upstream (*P* < 0.02). In the PSR, the average WSS magnitude was 19 ± 14 dyn/cm^2^ (Figs. 2 and 3). The area of maximal increased WSS was located slightly upstream to the maximum stenosis. All cases displayed a varying degree of increased WSS on a patch of the wall downstream from the stenosis away from the curvature of the vessel, more prominent in highly stenotic cases where the resulting blood flow jet hits the vessel wall, for example, cases 2 and 4 (Fig. 2). Compared to ideal laminar flow in a healthy vessel with vanishing components of the WSS in directions other than the axial direction of the bulk flow, the models in this study predict average axial WSS components different from the averaged WSS magnitude: in the stenosis the axial WSS component was 71 ± 49 dyn/cm^2^, and in the PSR the axial component was 8.0 ± 5.1 dyn/cm^2^.

### Pulsatile migration of zones of alternating shear vector directions

The temporal change of the WSS direction over the course of the cardiac cycle in response to the pulsatile flow conditions employed by our modeling approach was studied. Figure 2A–C shows three exemplary case types that are used to illustrate the highly complex and dynamic process that was predicted by our model. In the concentric stenosis depicted in Figure 2A (case 3), the stenosis is located close to the bifurcation and is followed by a tapered PSR (similar to the situation found in case 2). A concentric stenosis located distal to the bifurcation with a prestenotic dilatation and a tapered PSR up- and downstream is shown in Figure 2B (case 5) and can also be found in cases 1, 4, and 7 (Fig. 2D). Figure 2C shows a long and eccentric stenosis that spans a longer distance (case 6, similar to case 8).

The findings in the first two types of stenosis are similar: increased magnitude antegrade WSS vectors at the throat of the stenosis (area *s*, Fig. 2A and 2B) abruptly change direction to follow the recirculation of the blood flow that develops in the poststenotic dilatation (area *psr*), and change direction again downstream after reattachment of the flow in antegrade direction of the bulk flow (area *di*). The location of the lines separating these opposing shear directions shifts back-and-forth in a sweeping motion over the course of the cardiac cycle, moving more proximal during peak-systole and more distal during the diastolic phase of the pulse. In contrast, the line demarcating the reattachment and realignment of WSS in the main flow direction in the distal PSR is more distal during peak-systole and becomes more proximal during diastole. In case 6, a vortex of WSS vectors pointing into a retrograde direction is found in the proximal portion (circle a) and the midportion of the stenosis (circle b), where they change size and shape over the course of the pulse, in addition to another area of retrograde WSS that sweeps the distal portion of the stenosis in cyclic fashion (circle c, Fig. 2C).

### Rapid temporal change of regional shear stress distributions

This phenomenon of migrating zones of reversal of the WSS direction was further characterized by examining the temporal evolution of the axial WSS magnitude along a cutline through the stenosis throat (X-Y line, Fig. 3). In each case, we were able to identify a region along the chosen cutline that displayed a reversal of the direction of the WSS, typically between peak systole (t_2_) and the deceleration phase (t_3_) (cases 1–4 and 7), which is exposed to extreme directional changes of nearly 1412 ± 1037 dyn/cm^2^ in the short time between peak systole and deceleration phases of the cardiac cycle (green arrows in Fig. 3). The length of the portion of the chosen cutplane that shows a shear reversal was variable in length between 0.1 (case 5) and 1 mm (case 4).

### Complex temporospatial WSSG patterns

The spatial WSS Gradient (WSSG) magnitude, averaged over the course of the cardiac cycle, exhibited a visible increase in the area of the stenosis (Fig. 4A); in cases with concentric stenosis two distinct bands were found with increased axial component of the WSSG pointing in opposite directions (Fig. 4B). The previously defined three regions of interest (see insert Fig. 4C) were analyzed and the WSSG vectors were averaged spatially and temporally and the mean over all cases in this study was taken. The average WSSG magnitude at the throat of the stenosis was 1425 ± 1012 dyn/cm^3^, significantly increased from 8 ± 17 dyn/cm^3^ in the healthy CCA segment upstream (*P* < 0.002). In the PSR, the average WSSG magnitude was 140 ± 109 dyn/cm^3^ (Fig. 4C). The averaged axial WSSG components differed considerably from the averaged WSSG magnitude: in the stenosis the axial WSS component was 36 ± 273 dyn/cm^3^ (*P* < 0.002), and in the PSR the axial component was −6 ± 12 dyn/cm^3^ (*P* < 0.002).

The axial component of the spatial gradient of the WSSG along the vessel changes direction rapidly from positive to negative at the throat of the stenosis, corresponding to the peak axial WSS (line *a* to line *b* in Fig. 4D), followed by a second positive peak. WSSG at the stenosis ranges up to 24,000 dyn/cm^3^ in case 3, an approximate change of 50,000 dyn/cm^3^ occurs over 0.2 mm distance. The location of a pair of bands of negative WSSG followed by positive WSSG corresponds to the areas of increased WSS. A small band with low (near zero) WSSG separates the two (line *a*).

**Figure 5:**
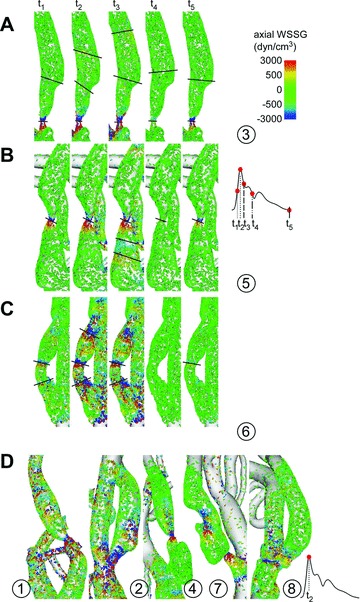
**(A–C)** Three example (case 3, 5, and 6) detailing the temporal evolution of the instantaneous wall shear stress gradient (WSSG) vectors at the stenosis and poststenotic region (PSR) during the cardiac cycle (reds points on pulse wave). Bands of acute reversal of this WSSG direction are demarcated by lines that demonstrate the stepwise changes of the location of these bands. **(D)** Instantaneous wall shear stress (WSS) vectors at peak-systole in cases 1, 2, 4, 7, and 8.

Similar to the complex patterns of the temporal change of the WSS direction over the course of the cardiac cycle WSSG exhibits its own dynamic. Figure 5A–C shows the same three case examples studied before, Figure 5D illustrates the distribution of WSSG vectors during peak systole for the remaining cases. In all three types of stenosis, a number of bands of acute changes of the WSSG direction were predicted that could be indicated by lines separating regions of WSSG vectors pointing in antegrade and in retrograde direction of the bulk flow (see lines in Fig. 5A–C). During systole these bands shift upstream compared to a more downstream location during diastole, and the number and magnitude of bands of positive and negative WSSG is increased during systole (Fig. 5B and 5C).

## Discussion

We examined in this pilot study the changes in flow patterns and the distribution of wall shear forces and their spatiotemporal derivatives in patient-based models of the carotid bifurcation in patients with CS, motivated by reports that stenosis in a vessel is associated with transient or even turbulent flow changes, high shear stresses in the stenosis, and low shear stress in certain regions proximal and distal to the stenosis (Cassanova and Giddens 1978). Previous analytic studies highlighted the effect of the eccentricity and shape of the stenosis on the flow pattern and shear stress distributions in the PSR (Steinman et al. 2000).

The intricate 3D geometry of the carotid bifurcation and stenosis is captured using the approach in this study with a level of detail that exceeds what has been reported thus far. The geometry of the vessel lumen serves as the dominant boundary condition and is a generator of a highly heterogeneous wall shear distribution on the vessel wall. The resulting predicted blood flow through the vessels and the stenosis and the resulting wall shear forces are sensitive to other boundary conditions. These include the pulsatility of the flow, the simulated material properties of blood, elasticity of the vessel, and viscoelastic properties of the blood components. The former two were addressed in this study; the latter two were ignored in our modeling approach. Our approach of employing high-quality CFD to a high-resolution reproduction of the vessel and stenosis geometry allows us to study the effect of this predominant boundary condition on the blood flow and elucidate the exact conditions to which blood is exposed within these patient-based lesions as opposed to idealized models. More readily available magnetic resonance imaging data that has been used to model flow in carotid bifurcations reproduces the anatomical detail of severe stenosis only poorly because of the limited ability of this technique to resolve flow-induced artifacts, and is less suited to provide high-quality geometric data as the basis of the modeling approach (Papathanasopoulou et al. 2003).

Interfacing between blood flow and the vessel wall, the EC serves as the principal sensor of mechanical forces exerted by the blood flow on the vessel wall. EC phenotype and cellular function are intimately linked to the local hemodynamic shear forces transmitted from the blood stream (Gimbrone et al. 1997; Nagel et al. 1999; Malek et al., 1999a, b). We sought to study the exact temporo-spatial pattern of shear stress beyond simply its magnitude in the light of the multitude of studies highlighting the response of vascular cells and blood components to temporal and spatial gradients of the shear in addition to the magnitude of the shear.

### Molecular effects of altered WSS

The generation of complicated flow dynamics, including recirculation and secondary flows in idealized stenosis and the alteration of a laminar flow regime found in a nonstenotic vessel, in and distal to the stenosis has been previously described (Cassanova and Giddens 1978), focusing on the dynamics and behavior of the blood flow itself rather than on the changes the latter imparts on wall shear forces. Our baseline WSS of 23 dyn/cm^2^ in the segment upstream of the stenosis is in agreement with the expected range of WSS in normal arteries. A multitude of in vitro studies of EC function evaluated the effects of low and high WSS of around 4 and 25 dyn/cm^2^, respectively, few studies studied the effects of very high shear (>100–200 dyn/cm^2^). Earlier studies by Fry describe a denudation of the canine aortic EC layer following imposition of WSS above 379 dyn/cm^2^ (Fry 1969). A more gradual experimental increase in WSS magnitude may allow the EC to develop the structural and functional adaptation needed to resist the even higher peak-systolic WSS values (>1000 dyn/cm^2^) seen in the 50–95% stenosis described here.

High WSS values greater than EC yield shear stress (Fry 1968) may further point to relationship between stenosis and exposure of blood to the subendothelial thrombogenic extracellular matrix. Activation of platelets in the flow through stenoses (Schirmer and Malek 2007a) and aggregation on the vessel wall, potentially leading to thrombosis, are known to be regulated by shear forces. A dysfunctional EC surface with partially denuded areas or one with increased EC–EC gaps may provide a pathological surface that may influence platelet adhesion and aggregation.

WSS changes within the stenosis itself and the PSR are highly complex and demonstrate significant variation throughout the cardiac cycle (Fig. 4) with interindividual variation reflecting the spectrum of geometry of the stenoses in this study. A zone of flow separation with reversal of the shear direction with downstream realignment is common to all lesions in this series, its location was observed to shift over time in cyclic fashion in all lesions studied here (Fig. 4A and 4B). EC in vitro demonstrate a differential response to specific biomechanical forces with different types of mechanical stimuli transduced into distinct phenotypes (Garcia–Cardena et al. 2001). We showed that in our simulations the predicted shear forces in CS lesions are altered in a fundamental way, providing a possible link between stenosed geometry and endothelial dysfunction in a vicious cycle of positive feedback. The zero WSS region identified between the distal stenosis and the PSR with convergent high WSS in opposing directions could have significant implications on local EC proliferation and turnover (Tardy et al. 1997; Nagel et al. 1999; Phelps and DePaola 2000; Schirmer and Malek 2007a).

Matrix metalloproteinases and their inhibitors play a central role in the remodeling of the arterial wall and the stability of fibrous caps covering atherosclerotic plaques. Although their distribution was found to be highly heterogeneous, a consistent two- to fivefold increase of the relative matrix metalloproteinase activity in four human carotid endarterectomy specimen was observed at the inflow proximal to the point of maximal stenosis in regions with expected elevation of WSS. (Choudhary et al. 2006) Reversal of the direction of WSS has been shown before without specific elaboration on the changes in WSSG at the neck of the stenosis and the PSR. (Stroud et al. 2002).

### Effects of altered WSSGs

Large spatial WSSGs in opposing directions were seen to develop in sharply delineated bands at the neck of the stenosis in all cases. Although our results indicate high WSSG in the range of 10^3^ dyn/cm3, data detailing the effect of WSSG in this range remain sparse. In an in vitro study by de Paola, the highest WSSG achieved was 600 dyn/cm^3^, one order of magnitude lower compared to our results, and this led to differential expression of numerous nuclear transcription factors (DePaola et al. 1992; Tardy et al. 1997; Nagel et al. 1999). Functional modulation of the ECs included reduced EC densities and increased mitotic rates up to 25% (DePaola et al. 1992) and permeability (Phelps and DePaola 2000) and decreased EC intercellular coupling and permanent gap-junction disruption (DePaola et al. 1999). The in vitro transendothelial albumin permeability increased by 5.5-fold for WSS with a spatial gradient compared to spatially uniform WSS (Phelps and DePaola 2000). Gertz and Roberts (1990) found shear stresses greater than 300 dyn/cm^2^ in stenosed coronary vessels; shear forces in this range can mechanically damage ECs and potentially strip them off of the vessel wall (Fry 1968). In another study, high WSSG of 240 dyn/cm^3^ led to increased proliferation of the EC in vitro, followed by migration and accumulation of the EC that culminated in EC detachment in vitro (Tardy et al. 1997).

We predict the presence of a sharply delineated band of low WSSG near zero limited by narrow bands of opposing WSSG directions at the neck of the stenosis, a result that has most likely not been uncovered in in vitro experimental setups because of the limitations of the possible stenosis geometry and flow regimes that were possible with these setups. A zone of convergence arises between opposing bands of WSSG with potential migration and subsequent accumulation in the middle is contrasted by zones of relative thinning of the EC density upstream and downstream to the stenosis. Large differences between systolic and diastolic flow regimes lead to significant variation of the WSS (Ku et al. 1985) during the pulsatile cycle. Stroud et al. postulate that the latter in conjunction with repetitive cycling loading and unloading mechanically weakens the plaque, increasing the likelihood of a plaque rupture (2002). We are showing that a cyclic change occurs over the pulse cycle, both of the magnitude and the spatial location of the areas of high WSSG both down- and upstream of the stenosis. Bao et al. (1999) showed that high temporal gradients of shear but not steady shear stress correlate with the expression of atherosclerosis-related genes in ECs and stimulation of endothelial and smooth muscle cells and exert a promitogenic effect on EC (White et al. 2001), possibly mediated by ERK1/2 pathway (White et al. 2005).

### Limitations of the approach

Our approach focuses on modeling the hemodynamic conditions within the vessel lumen and the shear stress on the vessel wall. These forces result in a multitude of effects on the morphology and function of the cells of the vessel wall and the particulate elements within blood but do not incorporate a feedback in the form of, for example, viscoelasticity of the vessel wall. Our reductionist approach also represents a snapshot in time, well after carotid stenosis has already progressed to a symptomatic lesion, and does not account for the complex multicellular autocrine and paracrine interactions among the various vessel wall cells and components. Other limitations of the current modeling technique include the assumption of a rigid wall and disregard for plaque composition and heterogeneity; it is accordingly not well suited at evaluating the tensile stresses within a vulnerable plaque. The influence of the external carotid artery and the intracranial collateral circulation was not considered in our approach, as we sought to limit the analysis to the area of the carotid bifurcation.

## Conclusion

In this series of patients with symptomatic carotid stenosis, we examined the abnormal flow pattern and wall forces around the stenotic area predicted by CFD simulation. These changes vary over the pulse cycle and give rise to temporal and spatial WSSGs, forming narrow bands of antegrade and retrograde WSSG next to areas of increased WSS. The magnitude and location of these regions of increased WSSG undergo cyclic changes over the cardiac cycle, exposing ECs in these areas to repetitive changes in direction and magnitude of the WSS and WSSG. We believe that these results provide data to guide further experimental studies and understanding of the hemodynamic component of the mutifactorial driving forces behind the progression of carotid disease.
